# Assessment of the Burden of Small Intestinal Bacterial Overgrowth (SIBO) in Patients After Oesophagogastric (OG) Cancer Resection

**DOI:** 10.1007/s11605-021-05177-w

**Published:** 2021-10-20

**Authors:** K.-V. Savva, L. Hage, I. Belluomo, P. Gummet, P. R. Boshier, C. J. Peters

**Affiliations:** 1 Department of Surgery and Cancer, St Mary’s Hospital, Imperial College London, 10thFloor QEQM Wing, London, W2 1NY UK; 2 Department of Gastroenterology, Imperial College Healthcare Trust, London, UK

**Keywords:** SIBO, Gastrointestinal surgery, Quality of life, GHBT

## Introduction

Small intestinal bacterial overgrowth (SIBO) is characterised by a change in the number/type of bacteria within the small intestine and is a common feature of patients who have undergone gastroesophageal reconstruction.^1^ Symptoms of SIBO are characterised as non-specific and range from bloating to malnutrition. The aim of the current study is to determine the prevalence of SIBO in oesophagogastric cancer (OGC) resected patients and to investigate the impact of this disorder on gastrointestinal (GI) symptoms and HRQoL.

## Methods

Patients who had previously undergone oesophagectomy (*n* = 30) and gastrectomy (*n* = 15) for gastroesophageal cancer, regardless of current GI symptoms, were recruited. Inclusion criteria were age ≥ 18 years, ≥ 1 year after surgery and free from disease recurrence at the time of assessment. Participants unable to provide informed written consent, suffering from liver disease, active infection, diabetes or had received antibiotic therapy within the previous four weeks, were excluded. A standard glucose hydrogen breath test (GHBT) using the GastroGastro + breath analyser was performed in all patients to assess SIBO occurrence. Current digestive symptoms were assessed in all patients using validated questionnaires evaluating overall digestive health and quality of life. Statistical analysis was performed using GraphPad Prism (version 7.0, La Jolla, CA, USA), Chi-squared tests and *T*-tests were used for univariate comparisons between GHBT( +) and ( −) patients responses. A *P*-value < 0.05 was considered to be statistically significant.

## Results

Of the 190 patients who were approached to participate in this study, 45 met the inclusion criteria (Table 1). SIBO is a significant clinical concern after foregut surgery as supported by the high incidence (73.5%) of SIBO( +) patients in the tested cohort. Rates of positive GHBT were equivalent in patients who underwent oesophagectomy (73.33%, *n* = 22) and gastrectomy (73.33%, *n* = 22). Likewise, time since surgery, chemotherapy, alcohol consumption, smoking, use of proton pump inhibitors, BMI and years from surgery did not significantly influence the data, suggesting that these variables were not confounding factors in the current study. Mean digestive symptoms scores reported by the EORTC-QLQ-C30, questionnaire, were not significantly different between GHBT( +) and GHBT( −) patients (Table 2). Within the EORTC-QLQ-C30 questionnaire, there was a non-significant trend towards greater ‘appetite loss’ amongst GHBT( +) patients (24.1 ± 31.9 vs. 9.1 ± 21.5; *P* = 0.160) (Table 2).

**Table 1:** Demographics of post GI surgery participants

	GHBT − *n* = 12	GHBT + *n* = 33	*P-value*
Sex (male:female)	10:2	27:6	> 0.999
Age	56.92 ± 16.87	70.94 ± 9.12	0.003^a^
BMI (kg/m^2^)	24.95 ± 5.65	24.43 ± 4.22	0.724 ^b^
PPI usage	5 (55.56)	17 (51.52)	0.079
Smoking	0	1 (3.23)	> 0.999
Alcohol usage	5 (55.56)	21 (72.41)	0.306
Chemotherapy	9 (81.8)	28 (84.85)	0.661
Interval from surgery (years)	7.59 ± 2.71	7.89 ± 3.5	0.945^b^
Surgical technique			
Two stage oesophagectomy	6 (50.00)	14 (42.42)	
Three stage oesophagectomy	2 (16.67)	8 (24.24)	
Subtotal gastrectomy	0	4 (12.12)	
Total gastrectomy	4(33.33)	7 (21.21)	> 0.999

Fisher’s exact test was used to determine the *P*-value, except for (a) which was determined by Kruskal–Wallis test, (b) by chi-square test and (c) by Mann–Whitney *U* test. *STDEV* standard deviation, *BMI* biomass index, *PPI* proton-pump inhibitors

**Table 2:** EORTC QLQ-C30

	All patients *n* = 43	GHBT ( +) *n* = 32	GHBT ( −) *n* = 11	*P* value^1^
Global health status	69.6 ± 19.4	69.1 ± 19.4	71.2 ± 20.2	0.227
Physical functioning	85.4 ± 17.4	83.5 ± 16.8	90.9 ± 18.7	0.057
Role functioning	84.1 ± 23.1	80.2 ± 25.2	95.5 ± 7.8	0.361
Emotional functioning	76.6 ± 25.0	74.5 ± 27.6	82.6 ± 14.7	0.111
Cognitive functioning	81.0 ± 21.4	78.6 ± 22.1	87.9 ± 18.4	0.221
Social functioning	79.5 ± 27.9	77.1 ± 29.6	86.4 ± 22.1	0.347
Fatigue	33.1 ± 27.1	35.4 ± 29.4	26.3 ± 18.1	0.339
Nausea and vomiting	11.9 ± 23.4	14.1 ± 24.4	6.1 ± 20.1	0.340
Pain	17.8 ± 23.4	18.8 ± 25.3	15.2 ± 17.4	0.665
Dyspnoea	20.2 ± 25.3	22.9 ± 26.1	12.1 ± 22.5	0.227
Insomnia	33.3 ± 29.1	36.5 ± 30.9	24.2 ± 21.6	0.234
Appetite loss	20.2 ± 30.1	24.1 ± 31.9	9.1 ± 21.5	0.160
Constipation	10.8 ± 22.7	10.4 ± 24.6	12.1 ± 27.1	0.832
Diarrhoea	23.3 ± 25.8	24.1 ± 25.7	21.2 ± 27.1	0.764
Financial difficulties	17.1 ± 30.5	21.4 ± 34.2	6.1 ± 13.5	0.159

Results are presented as mean ± standard deviation

^1^Comparison across different patient groups was performed by *t*-test

## Discussion

The current study provides valuable new insights for SIBO after surgery for OGC. The high rate of suspected SIBO in GHTB( +) patients suggests that the burden of this condition is under reported after OGC surgery.^1,2^ Patient reported outcomes suggest that the manifestations of SIBO are nonspecific and include a range of symptoms that overlap with other digestive disorders .^3^

GHBT was used to diagnose SIBO in this study. Two principal breath tests have been developed for the diagnosis of SIBO: GHBT and lactulose HBT, the latter requiring the administration of lactulose as opposed to glucose.^4^ The low sensitivity that is seen with both the GHBT and LHBT, with LHBT having a lower specificity and sensitivity than GHBT for SIBO diagnosis,^5^ would tend to result in a higher false negative rate. This indicates that SIBO may in fact be underdiagnosed in populations assessed by these methods. Potential reasons for false positive results include colonic fermentation gas production and rapid intestinal transit.^6^. The possibility of underestimating SIBO( +) patients by the use of HBTs further supports that SIBO is a significant concern upon OG resection, as SIBO prevalence might actually be greater than 73.5%.

In summary, this study (i) has demonstrated that SIBO does not exhibit specific clinical symptoms thus making its clinical diagnosis even more difficult and (ii) emphasised the need to determine appropriate guidelines for its assessment and treatment after OGC resection.
